# Review of Intranasal Active Pharmaceutical Ingredient Delivery Systems

**DOI:** 10.3390/ph17091180

**Published:** 2024-09-07

**Authors:** Ruslan Safarov, Olga Fedotova, Anastasia Uvarova, Mariia Gordienko, Natalia Menshutina

**Affiliations:** Department of Chemical and Pharmaceutical Engineering, Mendeleev University of Chemical Technology of Russia, Miusskaya pl. 9, 125047 Moscow, Russiauvarova.a.a@muctr.ru (A.U.);

**Keywords:** nasal medications, intranasal drug delivery systems, methods to improve API delivery

## Abstract

In recent decades, there has been an increased interest in the development of intranasal delivery systems for active pharmaceutical ingredients (APIs) not only for treating local nasal diseases but also for treating systemic diseases, central nervous system (CNS) disorders, and vaccine delivery. The nasal cavity possesses a unique set of anatomical characteristics for delivering active pharmaceutical ingredients, but there are several limitations that recent research in the field of the intranasal administration of APIs aims to overcome. For the effective delivery of nasal preparations, active pharmaceutical ingredients are incorporated into various micro- and nanosystems. Some of the most commonly encountered API delivery systems in the scientific literature include liposomal systems, polymer particles with mucoadhesive properties, in situ gels, nano- and microemulsions, and solid lipid particles. This article provides a review of research on the development of nasal preparations for treating local nasal cavity diseases (in particular, for antibiotic delivery), systemic diseases (analgesics, drugs for cardiovascular diseases, antiviral and antiemetic drugs), CNS disorders (Alzheimer’s disease, Parkinson’s disease, epilepsy, schizophrenia, depression), and vaccine delivery. The literature data show that active research is underway to reformulate drugs of various pharmacotherapeutic groups into a nasal form.

## 1. Introduction

Intranasal administration of drugs has been used for centuries for preventive purposes, but was mainly limited to the treatment of local diseases of the nasal cavity, such as rhinitis. The growing number of neurological diseases pushed forward research in the field of drug delivery by intranasal administration, and in 1991, William Frey patented a method for nasal delivery of drugs from the nose to the brain for the treatment of neurological diseases [1]. Since then, many interesting studies have been carried out in this area. Nasal drug delivery systems are being actively developed as an alternative to oral and parenteral administration. There is an increased interest in obtaining nasal drugs for systemic treatment [2], treatment of diseases of the CNS [3], and for vaccine delivery [4]. All this is due to the fact that the nature of the nasal mucosa provides a number of unique characteristics that contribute to the effective and convenient delivery of drugs. The nasal cavity is well vascularized. Thanks to this, drug molecules can be quickly transferred through one layer of epithelial cells directly into the systemic circulation without the hepatic and intestinal metabolism that occurs with oral administration [5]. This makes it possible to achieve a rapid therapeutic effect, especially for molecules with low molecular weight. However, it is necessary to introduce permeability enhancers in compositions containing high-molecular-weight drugs [6]. Nasal delivery may be suitable for drugs that are effective in small doses and have low oral bioavailability [7].

Advantages of nasal drug delivery [8]:

- Suitable for drugs that quickly degrade in the acidic environment of the stomach;
- Ensures rapid absorption of the drug and the onset of its action;
- Provides higher bioavailability of the drug, leading to lower required doses;
- Convenience and good patient compliance;
- Direct transport of the drug into the systemic circulation or central nervous system is possible;
- Direct delivery of vaccines to lymphatic tissues;
- Convenient for patients undergoing long-term therapy;
- Less risk of overdose;
- Non-invasive.

Limitations of nasal drug delivery [8]:

- The deliverable dose is limited to 25–200 µL;
- Difficulty in delivering high-molecular-weight APIs;
- Protective mechanisms (e.g., mucociliary clearance) may affect drug absorption;
- Topical enzymes in the nasal cavity may degrade some APIs;
- Local side effects may occur (mucous membrane irritation);
- Nasal congestion due to a cold may interfere with the effective delivery of the API.

**Figure 1:**
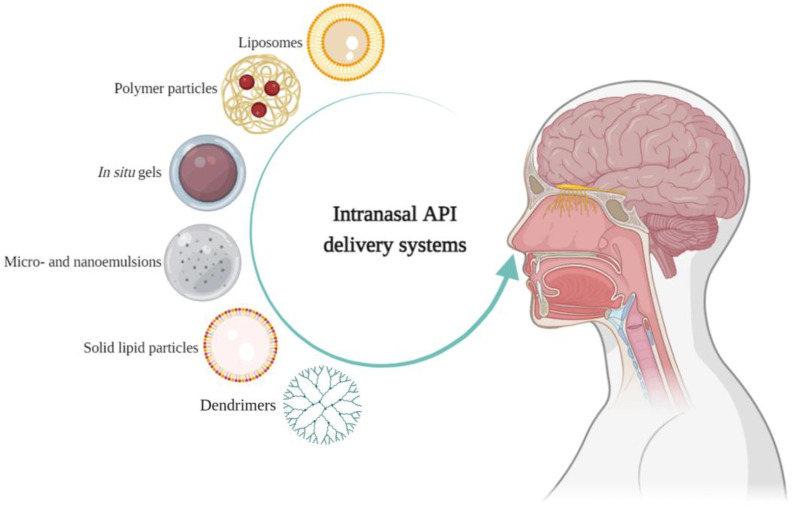
Drug delivery systems under investigation. Created with BioRender.com.

Typically, intranasally administered drugs are solutions, suspensions, gels, and emulsions. When developing formulations for intranasal delivery, much attention is paid to ensuring uniformity of dosage, stabilization of both the composition and the API, microbiological purity and a number of other quality aspects of the finished dosage form. To optimize and improve the adsorption of APIs during nasal administration, various delivery systems are being investigated and tested [9]. For example, active pharmaceutical ingredients can be incorporated into liposomal systems, polymer particles with mucoadhesive properties, in situ gels, nano- and microemulsions, solid lipid particles and dendrimers (Figure 1 and Table 1).

The purpose of this paper is to analyze and summarize the state-of-the-art in the field of nasal drug delivery. The article provides an overview of research of various intranasal delivery systems for the treatment of local and systemic diseases, diseases of the central nervous system, and for vaccine delivery.

**Table 1:** Intranasal API delivery systems.

Delivery System	Description	Refs.
Liposomes	Spherical vesicles consisting of one or several bilipid layers surrounding the aqueous phase. Hydrophilic drugs can be introduced into the aqueous phase, and hydrophobic drugs can be introduced into the bilipid layer of the liposome. Encapsulation of drugs in a liposomal system avoids degradation of the active pharmaceutical ingredient in the body and multidrug resistance.	[10]
Mucoadhesive polymer particles	They have the ability to adhere to the surface of mucous tissue, which leads to an increase in the concentration of the active pharmaceutical ingredient. This property allows for a reduction in the administered drug total dose.	[11]
In situ gels	Drug delivery systems that are used in the form of solutions or suspensions and are capable of phase transition in a certain place of the body under the influence of external factors such as temperature, pH, etc. These systems provide targeted release of active pharmaceutical ingredients and maintain them at a relatively constant concentration.	[12]
Micro- and nanoemulsions	Liquid-dispersed systems with a very fine distribution of droplets. These systems enable the delivery of hydrophobic drugs at a higher dose and have improved stability than conventional emulsions.	[9]
Solid lipid particles	They combine the advantages of delivery methods such as emulsions and liposomes; may have high capacity of active pharmaceutical ingredients and high API protection against the body environment factors.	[13]
Dendrimers	Highly ordered, branched polymer molecules with a symmetrically branched structure around a multifunctional central core. They have the ability to highly selectively encapsulate APIs.	[14]

## 2. Results and Discussion

### 2.1. Nasal Preparations for the Treatment of Local Diseases

The first barrier to the penetration of various bacteria and viruses is the nasal mucosa. The entry of pathogens into the nasal cavity can cause the development of rhinosinusitis, which is accompanied by symptoms such as nasal congestion, rhinorrhea, sneezing and itching [15]. Depending on the duration of symptoms, rhinosinusitis is classified as acute (lasting up to one month), subacute (from one to three months), chronic (more than three months) or recurrent (four or more repeating episodes of the inflammatory process during the year, with complete recovery in between) [16]. Until recently, it was believed that this disease did not have any serious consequences, but during the SARS-CoV-2 pandemic, the World Health Organization (WHO) declared rhinosinusitis a risk factor for patients with COVID-19 [17]. It is important to treat this pathology in a timely manner, as it can contribute to the development of a more dangerous disease.

Nasal administration of drugs is the most suitable method for treating diseases of the upper respiratory tract, allowing easy administration of a wide range of drugs. The most common dosage forms of nasal drugs are liquid dosage forms (solution, suspension or emulsion) for nebulization [18]. They contain one or more medicinal and auxiliary substance, homogeneously distributed, as a rule, in an aqueous environment. Popular drugs for the treatment of local diseases of the nasal cavity caused by allergic reactions or infections are antihistamines and corticosteroids [2]. Compared to oral administration, nasal preparations require lower doses of active substances. This reduces the risk of systemic side effects, such as drowsiness, which can occur with oral antihistamines.

Of particular interest is the intranasal administration of antibiotics [19]. Antibiotics are a group of drugs that act directly on bacteria to treat infectious diseases. Oral administration is the most common way to take antibiotics. However, the oral route of antibiotic administration can lead to numerous side effects [20,21]. Administration of antibiotics by the nasal route can provide precise treatment to the target site, significantly reducing the impact on other organs. For example, for the treatment of local nasal diseases (chronic rhinosinusitis), which last about 2 weeks, intranasal drugs can be used instead of oral antibiotics [22]. Intranasal administration of antibiotics is critical to reduce side effects, along with reducing the frequency of drug use and, importantly, minimizing the risk of resistance due to patient interruption during long-term treatment. Table 2 provides some examples of research on intranasal antibiotic delivery systems.

**Table 2:** Investigational nasal formulations for antibiotic delivery.

Antibiotic	Form	Therapeutic Indication	Refs.
Mupirocin	Solution	Chronic rhinosinusitis	[23,24,25]
Mupirocin and neomycin	Ointment	Staphylococcal rhinitis	[26]
Tobramycin	Spray	Bacterial rhinosinusitis	[27]
	Solution	Nasal polyposis	[28]
Vancomycin	Solution	Sinonasal polyposis	[29]
Ciprofloxacin	Drops, spray, gel	Chronic rhinosinusitis	[30]
	Microemulsion	*S. aureus* infection	[31]
	Gel in situ	Local infection	[32]
Levofloxacin	Gel in situ	Local infection	[33]

The authors of [31] developed a microemulsion for the intranasal delivery of ciprofloxacin for the treatment of infection caused by *Staphylococcus aureus*. Ciprofloxacin is a quinolone antibiotic with a broad spectrum of activity against many pathogenic bacteria, including *S. aureus*. To obtain a microemulsion, isopropyl myristate, polysorbate 80/ethyl alcohol (in a ratio of 2:1) and water in a ratio of 2.5%, 42% and 55%, respectively, were used. After this, 0.30% ciprofloxacin was added to the mixture. The penetration ability of the developed ciprofloxacin microemulsion was higher than that of pure ciprofloxacin.

The authors of [33] conducted an in situ study of a thermosensitive gel with levofloxacin. Levofloxacin belongs to a broad-spectrum antibacterial agent from the class of third-generation fluoroquinolones. As a result of the study, API concentrations were measured in the nasal mucosa and in blood plasma after intranasal and intravenous administration. It was found that the amount of levofloxacin in plasma was minimal after intranasal administration, indicating a reduced likelihood of adverse reactions. When the drug was administered to rats, the concentration of levofloxacin in the first 60 min in the mucous membrane of the anterior part of the nose after intranasal administration showed a greater value than after intravenous administration.

### 2.2. Nasal Drugs for the Treatment of Systemic Diseases

**Figure 2:**
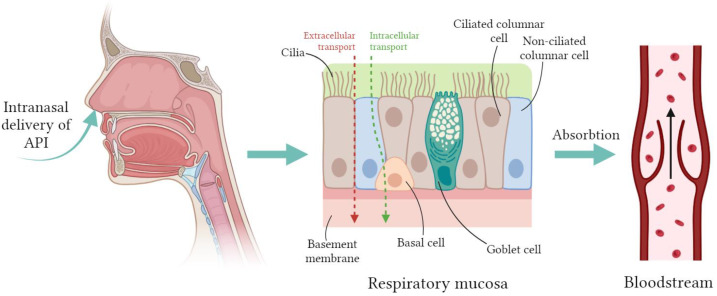
Schematic diagram of the mechanism of action of APIs for the treatment of systemic diseases with intranasal administration. Created with BioRender.com.

In addition to treating local diseases of the nasal cavity and paranasal sinuses, nasal medications can also be used to treat various systemic diseases. The use of intranasal delivery of systemic drugs is being studied as an analgesic for the treatment of cardiovascular diseases, for the prevention of infections, for the prevention of gag reflexes, etc. [34]. Researchers’ interest in developing such delivery systems is due to the advantages of intranasal administration, which were mentioned above. Nasal arterial blood supply is an essential factor for systemic absorption. Transport of APIs from the nasal epithelium directly into the bloodstream occurs mainly via intracellular and/or extracellular routes (Figure 2).

Let us take a closer look at the selected groups of systemic drugs for which research is being conducted.

#### 2.2.1. Painkillers

Painkillers are aimed at eliminating headaches and acute, moderate, severe, and chronic pain caused by surgery, injury or cancer. Cancer patients are the most in dire need of pain relief. In patients with cancer, pain can be divided into chronic and acute. Acute pain occurs spontaneously and is known as breakthrough pain. Painkillers to treat such cases must have a rapid onset of action and a sustained release. Typically, oral and parenteral analgesic solutions are used to treat breakthrough pain, but they have significant disadvantages: the initial effect of oral forms is achieved only within 30–45 min after administration, and the maximum effect is achieved within 1 h [35,36]. In the case of parenteral analgesics, the effect of the drug is approximately 5 min, but the invasiveness of administration requires the participation of trained people [36]. Intranasal administration of analgesics is a promising alternative, providing easy and rapid pain relief and improving the patient’s quality of life. A wide range of analgesic medications are being studied for intranasal administration, including morphine, fentanyl, buprenorphine, and others (Table 3).

**Table 3:** Investigations of intranasal systemic delivery.

API	Delivery System	Refs.
Morphine	Microparticles	[37]
Fentanyl	Spray	[38]
Buprenorphine hydrochloride	Solution	[39,40,41]
Ketamine	Solution	[42,43]
Sumatriptan	Gel in situ	[44]
	Nanoemulsion	[45]
	Powder	[46,47]
Zolmitriptan	Gel in situ	[48]
	Nanoparticles	[49]

According to the WHO recommendations, morphine is considered the standard analgesic drug for relieving pain in moderate-to-severe cancer. When morphine is administered orally, its bioavailability is only 20–30% [50]. When administered intranasally, the bioavailability of morphine is also low, amounting to only 10–30%, probably due to its very low lipophilicity. To increase bioavailability, preparations were developed in which mucoadhesive biopolymers were used as a carrier. In [37], the intranasal administration of chitosan microspheres with embedded morphine was studied. The bioavailability of the API composition of chitosan–morphine was 55%, and with the intranasal administration of a morphine solution, the bioavailability was 27%. In the same work [37], the bioavailability of a drug based on starch microspheres in combination with lysophosphatidylcholine was studied. Bioavailability for sheep was 75%. An increase in bioavailability when mucoadhesive biopolymers are used as API carriers may be associated with an increase in the residence time of the drug in the nasal cavity.

One of the most important clinical studies regarding intranasal morphine administration was to evaluate the pharmacokinetic profile and tolerability of a formulation consisting of morphine mesylate and chitosan [51]. Thirteen patients were enrolled in a randomized, complete crossover study with six single-dose morphine routes: intranasal morphine–chitosan 7.5 mg, 15 mg, and 30 mg; intranasal morphine (without chitosan, 15 mg); oral morphine sulfate (15 mg); and intravenous administration of morphine sulfate (10 mg). The absolute bioavailability of intranasal morphine with chitosan ranged from 60.4% to 82.3% at three dosage levels. The relative bioavailability of morphine in chitosan formulations compared to oral morphine sulfate was found to be more than 160%.

In [50], a study was conducted in which eleven patients were administered morphine solutions with the addition of oleic acid as an absorption stimulant. Improved bioavailability of morphine and increased mean residence time following nasal administration were demonstrated. In addition, patients reported a rapid onset of pain relief.

Unlike morphine, agents such as fentanyl and butorphanol can be absorbed effectively and rapidly through the nasal mucosa without the use of absorption enhancers. Fentanyl is a synthetic analgesic with high lipophilicity and low molecular weight, which facilitates direct penetration through the nasal mucosa. Fentanyl has been approved for marketing as a drug for the relief of postoperative pain, acute pain, procedural wound care pain, and breakthrough pain in patients with cancer. It is available in two different forms: an aqueous solution (Instanyl^®^) and a pectin-based mucoadhesive formulation (PecFent^®^). In a pharmacokinetic study conducted in nineteen cancer patients with breakthrough pain, fentanyl nasal spray showed rapid absorption through the nasal mucosa, reaching peak plasma concentrations within 12–15 min when administered in doses of 50, 100, and 200 μg [52].

In [38], a nasal pectin-based fentanyl spray was investigated. Compared to nasal fentanyl spray mixed with aqueous solutions, the pectin-based system reduces the maximum plasma concentration and provides prolonged release, which more closely matches the typical onset time of pain in cancer patients.

Triptans (selective serotonin receptor agonists) are another group of analgesics that are particularly effective for the treatment of migraine. Migraine is a type of headache usually characterized by moderate-to-severe pain that is throbbing and concentrated in only one part of the brain. During a migraine attack, pain-causing substances are released in the brain, causing cerebral blood vessels to dilate and stimulate nerves. Patients with recurrent migraine or cluster headache are traditionally prescribed oral tablet formulations of triptans, the most common of which are sumatriptan and zolmitriptan. However, their oral administration is often accompanied by a slow onset of action, and first-pass metabolism through the liver leads to low absolute bioavailability (14% for sumatriptan and 40–45% for zolmitriptan) [53,54].

Intranasal administration of sumatriptan and zolmitriptan is more effective and cost-effective, and these drugs are currently available in nasal forms. Their high lipophilicity facilitates passage through the nasal mucosa, which in practice allows for the administration of simple nasal solutions. Pharmacokinetic studies in healthy human volunteers have shown that the absorption of zolmitriptan administered as a nasal spray is very rapid, resulting in therapeutic concentrations in plasma within 2 min of dosing [55] and in the brain within 5 min [56]. Moreover, patients reported headache relief within 10–15 min after taking the drug [54]. To increase bioavailability and ensure prolonged release of sumatriptan, a thermosensitive gel was prepared in situ in [44]. The optimal composition of the gel was selected based on poloxamer 407 and poloxamer 188 using hyaluronic acid as a mucoadhesive polymer. Studies in sheep showed drug release (95.98%) within 6 h without histological or pathological changes in the sheep's nasal tissue.

#### 2.2.2. Drugs for the Treatment of Cardiovascular Diseases

Table 4 provides some examples of studies on the development of nasal drugs for the treatment of cardiovascular diseases.

As an alternative to parenteral therapy, ref. [57] developed a nasal drug for the delivery of metoprolol tartrate, used in the treatment of hypertension and angina. The drug was a microsphere based on sodium alginate (mucoadhesive biopolymer), the size of which ranged from 55 to 80 microns. The studies revealed that the maximum concentration of metoprolol in the blood plasma of rabbits and rats was higher after intranasal administration than after oral administration. In addition, the microspheres also provided a more sustained and controlled delivery of metoprolol tartrate compared to oral and parenteral administration.

**Table 4:** Investigational nasal drugs for the treatment of cardiovascular diseases.

API	Delivery System	Refs.
Metoprolol tartrate	Microspheres	[57]
	Gel in situ	[58]
Nifedipine	Solution	[59]
Carvedilol	Transfersomes	[60]
	Microspheres	[61,62]
	Gel in situ	[63]

Nifedipine is a calcium channel blocker often used to treat angina and hypertension. The authors of [59] conducted a crossover clinical study to investigate the optimal method of administering nifedipine for the rapid treatment of hypertension in six human volunteers. This study found that intranasal administration of nifedipine resulted in a lower peak blood concentration than that obtained with oral administration. Despite this, the mean serum concentration of nifedipine after 5 min was the highest (and remained the highest until the next 15 min) when administered intranasally.

Intranasal carvedilol has been studied for the treatment of hypertension and stable angina [61,62]. Microspheres of sodium alginate and chitosan (particle size 20–50 µm) were studied as potential delivery systems for carvedilol. Both nasal microsphere systems showed absolute bioavailability above 65%. It should be noted that the bioavailability of chitosan microspheres was slightly higher than that of alginate microspheres. Studies [61,62] showed that the mean residence time and half-life were two times higher compared to intravenous administration. Due to the high mucoadhesive ability of sodium alginate and chitosan, both dosage forms provided prolonged release of carvedilol.

#### 2.2.3. Antiviral Drugs

Table 5 provides some examples of nasal antiviral drugs.

**Table 5:** Investigational nasal antivirals.

API	Delivery System	Refs.
Acyclovir	Lipids	[64]
	Liposomes	[65]
Zidovudine	Nanoparticles	[66]
Darunavir	Solution	[67]

Acyclovir is an antiviral drug that is primarily used to treat the herpes simplex virus. Acyclovir is currently available in several dosage forms, which have serious limitations. Intranasal acyclovir is a promising strategy, but acyclovir is virtually impenetrable through the nasal mucosa. To improve the effectiveness of intranasal administration of acyclovir, liposomal delivery systems have been developed [65]. Liposomes with embedded acyclovir had higher permeability in the nasal cavity than pure acyclovir. This study [65] used fifteen rabbits, which were divided into three groups: one group received acyclovir liposomes in the form of a nasal gel, another group received acyclovir nasal gel, and the third group received an intravenous injection of acyclovir solution. The absolute nasal bioavailability of acyclovir, calculated over 8 h, was 60.7% for liposomal gel and only 5.3% for pure acyclovir.

Zidovudine is the first antiretroviral drug developed (for the treatment and prevention of HIV infection). Since its approval in 1987, it has become a key treatment for acquired immunodeficiency syndrome (AIDS). The work [66] describes the preparation and evaluation of nanoparticles based on a mixture of polylactide with polyethylene glycol (PLA-PEG) containing zidovudine. Due to the presence of PEG, the trapping efficiency of the API was increased. The relative bioavailability of nanoparticles based on the PLA-PEG mixture was 2.7 compared to nanoparticles based on PLA, and 1.3 compared to the drug in the form of an aqueous solution. Thus, PLA-PEG nanoparticles, due to the presence of PEG, increased the bioavailability of the API compared to its aqueous solution.

Another drug recommended by the WHO for the treatment and prevention of HIV infection is darunavir. The study [67] compared the biodistribution of darunavir at two different concentrations, high (25 mg/kg) and low (2.5 mg/kg), using two routes of administration: intravenous and intranasal. Compared to intravenous administration, intranasal administration demonstrated significantly better penetration of the API into the brain at both low and high concentrations.

#### 2.2.4. Antiemetic Drugs

Eliminating attacks of nausea and motion sickness with nasal medications is a desirable alternative to oral and parenteral medications. This is due to the need in acute situations for a faster onset of action, which can be achieved with intranasal administration. With oral administration, the absorption of drugs in the intestine can vary significantly due to impaired gastric motility associated with the pathological situation, while nasal administration guarantees a constant dosage. Table 6 provides some examples of nasal antiemetic drugs.

**Table 6:** Investigational nasal antiemetics.

API	Delivery System	Refs.
Metoclopramide hydrochloride	Gel in situ	[68,69]
	Microspheres	[70]
Ondansetron	Solid lipid particles	[71]
	Lipids	[72]
	Microspheres	[73]

Metoclopramide hydrochloride is a potent antiemetic that is effective in relieving nausea and vomiting associated with cancer therapy, pregnancy, and migraine. When administered orally, the bioavailability of metoclopramide varies greatly and ranges from 32% to 98%. In addition, the short half-life of metoclopramide suggests oral administration of the drug 3–4 times a day. Intranasal administration of metoclopramide is considered a good alternative to oral administration as it overcomes the problem of heterogeneous bioavailability. The authors of [69] obtained and studied an in situ gel with embedded metoclopramide for intranasal administration. The developed systems provided prolonged release of the drug in vitro for 8 h.

### 2.3. Nasal Drugs for the Treatment of Diseases of the Central Nervous System

The use of oral and parenteral methods for administering APIs in the treatment of neurological disorders does not allow effective delivery of drugs to the central nervous system. This is mainly due to the fact that there are barriers in the brain, primarily the blood–brain barrier (BBB), which protects the central nervous system from the penetration of blood cells, pathogens, mediators, and neurotoxic plasma components [74]. The BBB consists of endothelial cells tightly adjacent to each other. So-called tight junctions are formed between endothelial cells, the role of which is that they prevent the penetration of various undesirable substances from the bloodstream into the brain tissue. Tight junctions between endothelial cells block intercellular passive transport. In this case, the intercellular transport of substances is blocked both from the bloodstream to the brain tissue and in the opposite direction—from the brain to the blood [75]. A non-invasive way to bypass the BBB is to administer drugs through the nose. The nose is not only located in close proximity to the brain, but also contains special nerves, the olfactory and trigeminal nerves, which have a direct connection with the brain, independent of the BBB [76].

**Figure 3:**
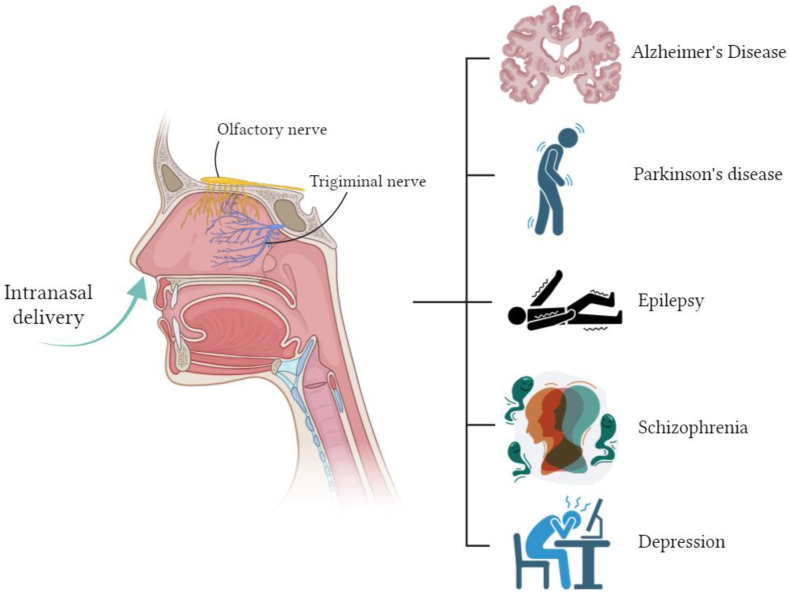
Intranasal delivery for the treatment of disorders and diseases of the central nervous system. Created with BioRender.com.

At the moment, there are many studies on intranasal drug delivery for the treatment of disorders and diseases of the central nervous system [2,9,77] (Figure 3).

Alzheimer’s disease is a slowly progressive neurodegenerative disease characterized by memory impairment and cognitive decline, which in turn affects behavior, speech, visuospatial orientation and the motor system, and is the most common form of dementia [78]. These problems are caused by the loss or destruction of neurons that are involved in cognitive functions in the brain. Oral medications are the most common treatment for Alzheimer’s disease, but their effectiveness is very limited. Table 7 provides examples of drugs for the treatment of Alzheimer’s disease.

In the study [83], chitosan nanoparticles with embedded piperine were obtained and studied. Piperine is a phytopharmaceutical with neuroprotective potential in Alzheimer’s disease. The effectiveness of nanoparticles with piperine was studied on 48 animals in which Alzheimer’s disease was induced. It was found that cognitive function was effectively improved as an injection of the standard drug (donpezil), but nanoparticles with piperine had the additional benefits of acetylcholinesterase inhibition and an antioxidant effect.

In [85], a gel based on poly-N-vinylpyrrolidone with covalently cross-linked insulin was obtained. When the resulting form was administered intranasally to mice, no changes or immunogenic response of the nasal mucosa were observed. In addition, an increase in insulin delivery to various brain regions and its biological activity was demonstrated compared to the administration of pure insulin.

The work [87] developed a method for delivering transfersomes with curcumin integrated into a nasal gel. Transfersomes are ultra-flexible vesicles with an aqueous core surrounded by a complex lipid bilayer. An in vivo study showed that the concentration of curcumin in the brain after intranasal administration was markedly higher than its concentration after intravenous administration. Curcumin transfersomes integrated into a nasal gel prolong mucosal contact time and release the drug in a controlled manner. The authors of [90] developed a liposomal form with donepezil for intranasal administration. These liposomal formulations were found to provide rapid and increased concentrations of donepezil in the brain. The research results showed that the bioavailability of the resulting form was doubled compared to the oral and parenteral routes of administration. In a study [94], a hybrid intranasal delivery system was obtained, including a nanosuspension of resveratrol as an API and deacetylated gellan gum. Deacetylated gellan gum is used as a gelling matrix in situ (in the nasal cavity) to increase residence time and improve absorption of the API. The results of studies [94] showed that with intranasal administration of the obtained form, the maximum concentration of resveratrol in the brain was 2.88 times higher than with intravenous administration of the standard form.

Parkinson’s disease is the second most common neurodegenerative disease, affecting 1.5% of the world’s population over 65 years of age [96]. Parkinson’s disease is characterized by progressive degeneration of the nigrostriatal dopaminergic system, which causes a loss of dopamine. Symptomatically, Parkinson’s disease is characterized by impaired motor function (slowness of movement, tremors, rigidity, and loss of balance) and other complications, including cognitive decline, mental disorders, sleep disturbances, pain, and sensory disorders [96]. Current treatment strategies for Parkinson’s disease primarily focus on relieving motor symptoms by increasing dopamine levels in the CNS or stimulating dopamine receptors. The most common treatment is oral levodopa. However, its long-term administration leads to serious side effects [97]. Thus, new approaches are needed that can increase the effectiveness of treatment for Parkinson’s disease. Table 8 provides examples of investigational nasal medications for the treatment of Parkinson’s disease.

In a study [98], chitosan nanoparticles with selegiline were obtained as an API for intranasal administration. Studies in rats showed that selegiline concentrations in the brain and plasma were 20 and 12 times higher, respectively, after intranasal administration than after oral administration.

Chitosan nanoparticles loaded with pramipexole dihydrochloride were obtained in [107]. In pharmacodynamic studies, the results showed an improvement in motor functions in a group of rats that received intranasal administration of the resulting nanoparticles, compared with pramipexole nasal solution or oral tablets.

Epilepsy is a chronic neurological disease that manifests itself in the body’s predisposition to the sudden onset of seizures [108]. Table 9 provides examples of nasal medications being studied for the treatment of epileptic seizures.

Lamotrigine is widely used as an antiepileptic drug [115]. Due to poor solubility in water, it has low effectiveness when administered orally. In [110], PLGA-based nanoparticles loaded with lamotrigine were obtained. In vivo studies were conducted on rats, and the delivery efficiency of lamotrigine nanoparticles was more than 120%. The maximum concentration of APIs in the brain was found to be 3.82 μg/mL 1.5 h after intranasal administration, while after oral administration, it was 1.4 μg/mL after 1.5 h.

Schizophrenia is a severe mental disorder that affects approximately 20 million people worldwide [116]. People with schizophrenia may suffer from positive (delusions, auditory hallucinations) and/or negative symptoms (social isolation, disorganized speech, inability to concentrate). Symptoms of schizophrenia can be effectively suppressed with atypical antipsychotics [117]. Table 10 presents some studies on the development of drugs for the intranasal delivery of atypical antipsychotics.

In [119], a liposomal delivery system for quetiapine fumarate was studied. Quetiapine fumarate has an oral bioavailability of 7–8% due to its low solubility in water. The use of liposomes as a delivery system for quetiapine fumarate for intranasal administration can increase the bioavailability of the drug by up to 32%. In a study [120], a nanoemulsion of asenapine maleate was prepared with the addition of the mucoadhesive polymer Carbopol 971 to increase the residence time on the nasal mucosa. The maximum concentration in the brain of asenapine maleate was 284.33 ± 5.5 ng/mL 1 h after intranasal administration, while with intravenous administration, it was 79.86 ± 8.2 ng/mL 3 h after administration.

The authors of [127] obtained chitosan nanoparticles loaded with risperidone. The developed composition of chitosan nanoparticles showed a significantly higher release of the API (81%), and its bioavailability was increased up to three times compared to the conventional dosage form in the form of a solution when delivered nasally.

Depression is a mental disorder that is characterized by emotional disturbances and can affect a person’s thoughts, behavior, and physical well-being [128]. Antidepressants are drugs that are used primarily for the treatment of depression and affect the level of neurotransmitters, particularly serotonin, norepinephrine, and dopamine. Intranasal delivery as a promising treatment for depression has been explored with several antidepressants. Table 11 presents some studies on the development of drugs for the intranasal delivery of antidepressants.

Thus, in [129], the intranasal delivery of agomelatine in the form of a gel in situ was studied, which had a sol–gel transition temperature of 31 °C, a mucociliary transport time of 27 min, and a release after 1 h of 46.3%, after 8 h—70.9%. A pharmacokinetic study of the gel revealed a 2.7-fold increase in the concentration of APIs in the rabbit brain compared to oral administration. The authors of [136] developed and studied a nanoemulsion containing paroxetine. Paroxetine is a selective serotonin reuptake inhibitor and is used to treat depression and anxiety problems. The results of a study of paroxetine nanoemulsion showed an increase in penetration by 2.57 times compared to a paroxetine suspension administered orally. Behavioral studies (forced swimming test and locomotor activity test) were conducted on rats to study the therapeutic effect of the resulting composition. Treatment of depressed rats with paroxetine nanoemulsion administered intranasally significantly improved behavioral activity compared with paroxetine suspension administered orally. In [135], chitosan nanoparticles loaded with venlafaxine were obtained for intranasal delivery. Venlafaxine is a dual-acting antidepressant (serotonin and norepinephrine reuptake inhibitor). Chitosan nanoparticles were prepared by ionic gelation of chitosan with sodium tripolyphosphate and freeze drying. Venlafaxine was dissolved in a chitosan solution at a ratio of 1:1 before adding sodium tripolyphosphate. The concentration ratio of venlafaxine in brain tissue and blood plasma 0.5 h after delivering the intravenous administration was 0.0293; for the intranasal administration of venlafaxine, it was 0.0700; and for the intranasal administration of chitosan nanoparticles with venlafaxine, it was 0.1612. The research results showed that chitosan nanoparticles with venlafaxine have faster API transport and a higher percentage of direct transport (80.3%).

### 2.4. Nasal Formulations for Vaccine Delivery

Most pathogens that cause severe illness (e.g., influenza, meningitis, coronavirus infection, and measles) enter the body through the nasal cavity. The nasal cavity has good anatomical characteristics and immune potential to combat infectious agents. Parenteral delivery of vaccines is the most common method of immunization, but can only have a systemic effect. Compared to parenteral delivery, intranasal delivery of vaccines can provide both systemic effects and induce local immunity. In addition, the nasal cavity is a highly vascularized area that allows for non-invasive vaccine delivery. Intranasal delivery of vaccines is a promising alternative to vaccinations and is suitable for mass vaccination [140].

Mucous membranes are endowed with powerful mechanical and chemical protection factors. The mechanisms of innate and adaptive immunity protect these surfaces, and therefore the internal environment of the body, from the potentially damaging effects of environmental factors, particularly infectious ones [141]. The secretion of the nasal mucosa contains various types of immunoglobulins, such as IgG, IgA, IgE, and IgM. When the vaccine is delivered to the nasal cavity, it stimulates the production of local secretory antibodies IgA and IgG [142].

The live attenuated influenza vaccine FluMist^®^ was first approved in 2003 for the treatment of people aged 5 to 49 years [143]. FluMist^®^ is an intranasally administered trivalent seasonal influenza vaccine containing three live influenza viruses: two type A viruses (subtypes H1N1 and H3N2) and one type B. In 2012, the Food and Drug Administration (FDA) approved FluMist Quadrivalent, a product containing two type A and two type B viruses, for use in individuals aged 2 to 49 years.

The success of FluMist^®^ and the demand for more effective vaccines against many different diseases have inspired the scientific community to conduct research on intranasal vaccine delivery [4].

Table 12 shows some research work on the development of nasal vaccines.

Intranasal vaccines require a carrier (adjuvant) to ensure antigen delivery and high efficiency with an immunostimulating effect [162]. As carriers, as a rule, biodegradable, non-toxic, and biocompatible compounds with immunostimulating properties are used. These include, for example, chitosan and its derivatives, hyaluronic acid, and sodium alginate [163,164].

The work [148] studied the production of a plasmid vaccine against hepatitis B using chitosan and human serum albumin as a carrier. Studies in mice showed that intranasal vaccinations induced a strong systemic and local immune response. The authors of [161] studied the production of nanoparticles loaded with influenza A virus antigen (PR8 subtype) based on chitosan or its water-soluble derivative trimethyl chitosan, with or without coating with sodium alginate. It was found that after intranasal administration, trimethyl chitosan-based nanoparticles caused a weaker immune response compared to chitosan-based nanoparticles. It was also found that sodium alginate-coated nanoparticles can induce a stronger immune response compared to uncoated nanoparticles, especially for trimethyl chitosan-based nanoparticles.

At the end of 2019, an outbreak of infection caused by the SARS-CoV-2 virus was registered in Wuhan (China). In 2020, the WHO declared a pandemic. The SARS-CoV-2 virus causes coronavirus infection (COVID-19), which is an acute respiratory disease and can occur in both mild and severe forms [165]. All licensed COVID-19 vaccines are administered parenterally (intramuscularly), which is ineffective for developing mucosal immunity. Intramuscular injections cause a systemic humoral response to the vaccine, which leads to the formation of first secretory immunoglobulin IgM and then IgG [166]. However, with respiratory viruses such as SARS-CoV-2, the mucosal immune system is the first line of defense, with the mucosal immune response causing the formation of secretory immunoglobulin IgA. This means that systemically vaccinated individuals are susceptible to SARS-CoV-2 infection through the upper respiratory tract. Intranasal vaccine delivery can induce both mucosal and systemic immune responses [166]. Nasal vaccination can not only provide protection against infection, but also prevent its spread.

In [154], intranasal vaccines were developed using PLGA-based microspheres loaded with peptides and oligonucleotides as carriers. The resulting vaccine was studied on rhesus monkeys. Clinical symptoms and viral infection were assessed in comparison to a control group and showed that vaccinated macaques had less infection and clinical symptoms. The authors of [157] studied the immunogenicity of the receptor-binding domain of the SARS-CoV-2 spike glycoprotein loaded into trimethyl chitosan nanoparticles. The results showed that intranasal delivery of the resulting vaccine to mice induces strong local mucosal immunity, as evidenced by the presence of IgG and IgA immunoglobulins. In addition, mice administered intranasally with this immunogen platform developed strong systemic antibody responses, including serum IgG, IgG1, IgG2a, IgA, and neutralizing antibodies.

### 2.5. API Carriers for Intranasal Delivery

In order to optimize and improve the adsorption of APIs during nasal administration, various delivery systems are being researched and tested. Polymer-based micro- and nanoparticles, in situ gels, nano- and microemulsions, solid lipid particles, and liposomes are innovative and the most popular methods of delivering APIs.

**Figure 4:**
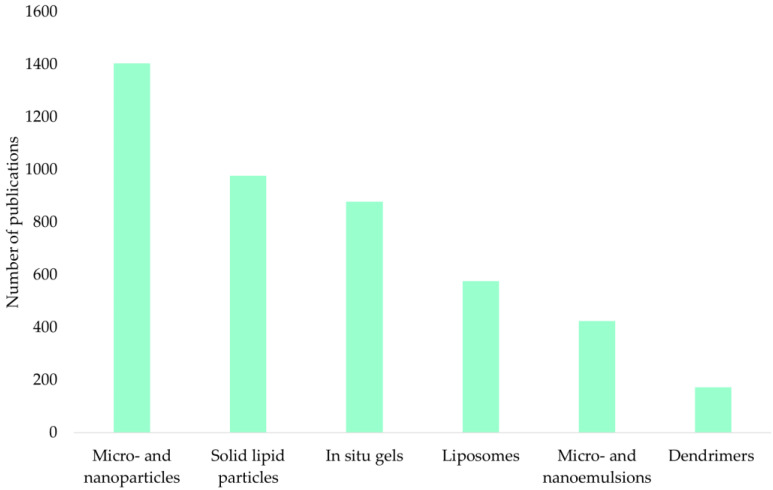
Number of publications for the considered intranasal API delivery systems according to the ScienceDirect Internet resource.

Figure 4 shows an analysis of the ScienceDirect scientific publication database for the keywords “intranasal microparticles”, “intranasal nanoparticles”, “intranasal gels in situ”, “intranasal microemulsions”, “intranasal nanoemulsions”, “intranasal liposomes”, “intranasal solid lipid particles”, and “intranasal dendrimers” for the period 2000–2023.

The presented diagram shows that micro- and nanoparticles (33%), solid lipid particles (23%), and in situ gels (21%) are of the greatest interest.

#### 2.5.1. Polymer Micro- and Nanoparticles

Polymer micro- and nanoparticles are solid porous particles in which the API is encapsulated or chemically bound to a polymer matrix. These delivery systems provide sustained/controlled release of the API, are biodegradable and biocompatible, and are inexpensive to manufacture [167]. To obtain particles, both natural and synthetic polymers are used (Table 13).

**Figure 5:**
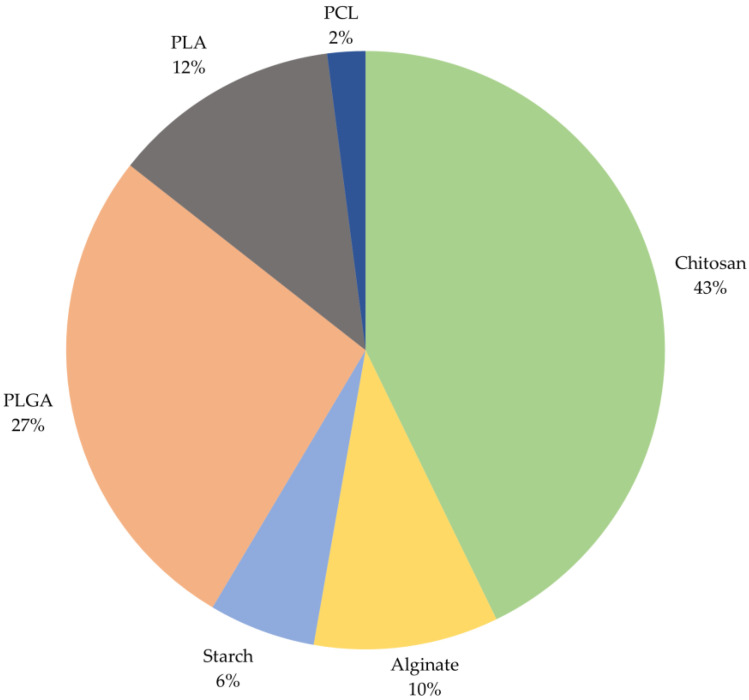
The ratio of polymer use based on an analysis of the ScienceDirect database of scientific publications.

As a result of the ScienceDirect scientific publication database analysis for the above-mentioned period, the frequency of implementing various polymers to obtain micro- and nanoparticles was determined (Figure 5).

Chitosan is of the greatest interest for obtaining particles as carriers. The advantages of chitosan over other polymers have been repeatedly demonstrated in a number of studies. For example, in [70], microspheres were obtained based on chitosan, bovine serum albumin, and sodium alginate containing metoclopramide hydrochloride. The results of the study showed that the highest loading of metoclopramide hydrochloride was achieved in chitosan microspheres and is equal to 91.95%.

Chitosan is the second most common polysaccharide in nature and is a cationic heteropolymer obtained from chitin, a natural polysaccharide that is the main component of the exoskeleton of arthropods and is part of the cell walls of fungi, a number of bacteria, and blue-green algae [168]. The physical and chemical properties of chitosan depend on its molecular weight and the degree of deacetylation. High-molecular-weight chitosan has low solubility in neutral aqueous solutions, which limits its use. A study [169] examined the effect of chitosan molecular weight on the characteristics of methotrexate-loaded chitosan microspheres. Microspheres consisting of low-molecular-weight (40 kDa), medium-molecular-weight (480 kDa), and high-molecular-weight (850 kDa) chitosan with the same degree of deacetylation (96%) were obtained by spray drying. The results of the study showed that microspheres with low-molecular-weight chitosan have better flowability and the highest bulk density, but have weak adhesion. Microspheres of medium-molecular-weight chitosan showed the strongest adhesion to the surface of the mucous membrane. Microspheres with high-molecular-weight chitosan exhibited lower adhesion and lower API release rates than medium-molecular-weight chitosan.

Chitosan has mucoadhesive properties, that is, the ability to adhere to mucous membranes due to electrostatic interactions, as well as bonds formed between the functional groups of chitosan and the molecules of the mucous membrane. In an acidic environment, the amino groups of chitosan are positively charged and, thus, can interact with negatively charged mucin molecules in the mucous membrane, which leads to mucoadhesion and promotes the release of APIs [149].

PEGylated nanoparticles can also be considered as promising API delivery systems for intranasal administration [170,171]. The article [171] shows that PEGylated nanopar-ticles were non-mucoadhesive, and hence displayed mucus-penetrating properties.

#### 2.5.2. Solid Lipid Particles

Solid lipid particles (SLPs) are dispersed systems consisting of a liquid dispersion medium and a solid dispersed phase. The main components of SLPs are water, lipids, and surfactants. Fatty acids, waxes, and esters of glycerol are used as lipids. Additionally, excipients (gelling agents, mucoadhesive agents, permeability enhancers, etc.) can be added to SLP-based systems [172]. SLPs can be coated with polyethylene glycol or its derivatives.

SLPs are obtained using high-pressure homogenization, solvent diffusion, emulsification or solvent evaporation methods [173]. SLPs protect the APIs from the action of enzymes in the nasal cavity and prevent its premature metabolic breakdown. In addition, they are considered to have low toxicity to humans due to the absence of toxic organic solvents during their production [173]. However, these systems have disadvantages such as poor storage stability, which can lead to particle aggregation, phase separation, and cause premature release of the APIs [172].

In the article [71], ondansetron-embedded SLPs were obtained for the treatment of postoperative nausea and vomiting caused by chemotherapy. SLPs were obtained by solvent diffusion using lecithin and co-surfactant Poloxamer 188 as a surfactant. Glycerol monostearate was used as a lipid material. The authors stated that when conducting in vitro release experiments, biphasic behavior was observed, which consisted of an initial rapid release of ondansetron from the particle surface, followed by a slow-release phase associated with the diffusion of the APIs from pores on the particle surface. Additionally, the study found that the resulting formulation was stable for 3 months.

The authors of [84] obtained and studied SLPs with incorporated piperine for the treatment of Alzheimer’s disease. SLPs based on glyceryl monostearate and epicuron 200 were obtained by the emulsification–solvent diffusion method. The authors conducted studies on rats of a pure piperine preparation, an SLP-based delivery system with an embedded drug, and an SLP-based delivery system with an embedded piperine after 3 months of storage. The results of the study demonstrated that the maximum concentration (C_max_) of piperine in the brain for SLP-based delivery systems is achieved 3 times faster than for pure piperine (60 min and 180 min, respectively). The C_max_ for SLP-based delivery systems is 2.5 times higher than the C_max_ of pure piperine. Thus, the authors demonstrated the effectiveness of using intranasal delivery systems based on SLPs, and demonstrated their stability for 3 months.

#### 2.5.3. In Situ Gels

In situ gels are an API delivery system that can transition from a liquid to a gel state under the influence of certain factors [9]. In the field of nasal API delivery, the use of in situ gels represents a promising approach to control the release and delivery of active substances into the nasal cavity. In situ gels can be divided into several types [174]:

- Thermosensitive systems, which demonstrate a phase transition at temperatures in the range of 25–37 °C;
- Ion-sensitive systems, which demonstrate a phase transition due to reactions with ions present in the nasal mucosa.

Examples of temperature-sensitive and ion-sensitive systems, as well as reactants responsible for the phase transition, are presented in Table 14.

To obtain temperature-sensitive systems, so-called “Pluronics” are widely used, having the trade names Pluronic and Poloxamer with a three-digit code indicating the molecular weight of the polyoxypropylene core and the percentage of polyoxyethylene in the polymer [176]. In aqueous solutions, with increasing temperature, Pluronics form micelles in order to reduce the free energy of the solution. Some of the most popular Pluronics for producing in situ gels are Pluronic F-127 and Poloxamer 407, which are biocompatible nonionic block copolymers with thermosensitive properties.

Some chitosan derivatives are capable of undergoing a phase transition with a change in temperature. For example, the authors of [144] developed a thermosensitive hydrogel based on *N*-[(2-hydroxy-3-trimethylammonium) propyl] chitosan chloride, which is a cationic derivative of chitosan. This composition is in a liquid state at room temperature, and it turns into a gel state at body temperature.

In situ ion sensing systems can be obtained using gums such as gellan and xanthan. Gellan gum is an extracellular anionic water-soluble polysaccharide produced by the bacteria *Sphingomonas elodea*. Xanthan gum is a natural polysaccharide formed as a result of the fermentation of the Gram-negative bacterium *Xanthomonas campestris* [12]. Gel formation is carried out by forming a complex with cations (sodium and calcium) present in the nasal mucosa.

Carbopol^®^ is a high-molecular-weight polyacrylic acid polymer that turns into a gel when pH increases. The acidic nature of this polymer can cause irritation, so hydroxypropyl methylcellulose (HPMC) is added to reduce its concentration and increase viscosity.

The authors of [101] prepared and studied in situ nasal gels with rasagiline mesylate. Nasal gels were prepared using various polymers such as hydroxypropyl methylcellulose, Carbopol^®^ 934, and sodium alginate. Studies have shown that a formulation containing sodium alginate provides better controlled release of the APIs than other formulations.

In [32], a thermosensitive gel in situ with ciprofloxacin was studied. One of the main problems of intranasal administration is the residence time of the drug in the nasal cavity. The temperature-sensitive in situ gel, due to its increased viscosity and mucoadhesion, can provide complete absorption and prolonged release of the API. The results of the study showed that the concentration of ciprofloxacin in the mucous membrane of both the anterior and posterior parts of the nose after intranasal administration was higher compared to intravenous administration. This made it possible to reduce the concentration of the antibiotic by 41 times compared to the drug for intravenous administration and avoid side effects.

#### 2.5.4. Micro- and Nanoemulsions

Microemulsions are dispersed systems consisting of a hydrophilic phase, a lipophilic phase, surfactants, and co-surfactants. Microemulsion droplets have sizes ranging from 10 to 200 nm. Microemulsions differ from conventional emulsions in the absence of turbidity. By using microemulsions, improved solubility and hence better stability, longer shelf life and increased bioavailability for poorly soluble APIs can be achieved [177].

Microemulsions are prepared by adding lower alcohols to water-in-oil (w/o) or oil-in-water (o/w) emulsions. In this case, lower alcohols act as co-surfactants and are responsible for reducing the interfacial tension between the aqueous and oil phases, ensuring the formation of a microheterogeneous system. Depending on the type of microemulsion, amphiphilic molecules are oriented in a certain direction. In the o/w system, the non-polar part of the molecules is directed into the dispersed phase; in w/o systems, this is vice versa. o/w systems are especially interesting for drug delivery, since the hydrophobic drug is easily dissolved in the internal oil phase of the microemulsion and is better transported and absorbed into the bloodstream due to the external aqueous phase. Highly lipophilic drugs are particularly suitable for microemulsions as drug delivery systems [177].

In contrast to microemulsions, nanoemulsions can be characterized as emulsions with a narrower droplet size distribution, lying in the range of 0.1–100 nm. Nanoemulsions are thermodynamically unstable disperse systems. As with microemulsions, o/w systems are especially important for API delivery systems. To ensure stability in the production of nanoemulsions, as well as for microemulsions, surfactants and co-surfactants are used as stabilizers [178].

For micro- and nanoemulsions of the o/w type, the solubility of the APIs in the oil phase is extremely important. The work [79] assessed the solubility of rivastigmine hydrochloride in oils (Capmul^®^ MCM, sunflower oil, fish oil, almond oil, olive oil, castor oil, Til oil, coconut oil, and Kalonji oil), surfactants (Cremophor^®^ EL, Cremophor^®^ RH 40, Capryol^®^ 90, Labrafil^®^ M, Labrasol^®^, and Tween^®^ 80) and co-surfactants (Captex^®^ 200-P, PEG-400, sorbitan sesquioleate, and Transcutol^®^ P). Among the selected oils, rivastigmine hydrochloride had the highest solubility in Capmul^®^ MCM (80 ± 2.64 mg/mL). Therefore, Capmul^®^ MCM was chosen as the oil phase. Among surfactants, rivastigmine hydrochloride showed the highest solubility in Tween^®^ 80 (45 ± 2 mg/mL). Therefore, Tween^®^ 80 was chosen as a surfactant. Tween^®^ 80 belongs to the class of nonionic surfactants and is widely used because it is less toxic compared to ionic surfactants. Among co-surfactants, Transcutol^®^ P showed the highest solubility, which is 60 ± 1.5 mg/mL. Therefore, Transcutol^®^ P was chosen as a co-surfactant. Transcutol^®^ P has the ability to form transparent and stable nanoemulsions.

To increase the residence time on the nasal mucosa, mucoadhesive polymers are added to some nanoemulsions. For example, in [120], Carbopol^®^ 971 was added to the nanoemulsion. The delivery of asenapine maleate was studied. Asenapine maleate is used to treat schizophrenia and has very poor water solubility and a high first pass effect, such that the final bioavailability is less than 2%. The research results showed that the maximum API concentration in the brain with the intranasal administration of a mucoadhesive nanoemulsion increased from 79.86 ± 8.20 ng/g to 284.33 ± 19.5 compared with the intravenous administration of a nanoemulsion.

#### 2.5.5. Liposomes

Liposomes are spherical vesicles with a hydrophilic core and a shell consisting of one or more phospholipid bilayers. A key advantage of the liposomal delivery system is the ability to deliver both hydrophilic and lipophilic (hydrophobic) APIs. Hydrophilic APIs dissolve in the liposome core, while lipophilic APIs dissolve in the lipid bilayer [179].

The most common method for producing liposomes is the thin film hydration method. This method involves dissolving phospholipids and lipophilic ingredients in an organic solvent, such as chloroform, and then evaporating it under reduced pressure to obtain a thin lipid film. When an aqueous phase is added to the resulting thin lipid film under intense mechanical action, liposomes are formed.

In [109], using the thin film hydration method, liposomes with incorporated lamotrigine were obtained. The composition was prepared using Phospholipon^®^ 90 G, cholesterol, and Tween^®^ 80 as starting ingredients. The study showed that the resulting liposomes with lamotrigine have higher bioavailability than the suspension with lamotrigine. In addition, a toxicity study showed that this composition is safe for intranasal delivery.

The work [122] investigated the preparation of a liposomal delivery system for risperidone. Risperidone is a drug with low molecular weight and high lipophilicity, due to which its bioavailability is low. To increase bioavailability, risperidone was introduced into liposomes. Liposomes consisting of soya phosphatidylcholine and cholesterol were prepared by thin film hydration. The authors of the study found that the maximum concentration of APIs in the brain, when liposomes were administered, was two times higher and four times faster than when the pure substance was administered.

#### 2.5.6. Dendrimers

Dendrimers are nanosized molecules with a symmetrical and branched structure. The physical and chemical properties of dendrimers depend directly on their three-dimensional structure. This structure consists of a multifunctional central core in which other molecules can be trapped, branched branches emanating from the central core, and outer surface groups. Dendrimers are characterized as macromolecules that are predictable, controllable, and reproducible with great accuracy, having symmetrical channels and pores in the branched structure of macromolecules. Dendrimers are capable of highly selective encapsulation of various substances and, accordingly, can be used for various purposes, including intranasal delivery. The encapsulation of guest molecules is driven by noncovalent interactions (ionic, H-bonding, and van der Waals interactions) and can be tailored for various drugs at the same time [180].

Dendritic polymers with their regular and well-defined unimolecular architecture, which can be further chemically modified at either the core (to increase hydrophobicity) or the shell (to increase hydrophilicity), are currently attracting interest as so-called dendritic nanocarriers for applications in drug solubilization and delivery [181].

In the article [182], the authors explore the preparation of dendritic polyglycerol-derived nano-architectures that can be used for the intranasal delivery of APIs. The article [183] presents the results of studies of polyamidoamine dendrimers (PAMAM) with amino groups on the surface for the delivery of haloperidol to the brain after intranasal and intraperitoneal administration. To obtain the drug, PAMAM dendrimer, ethanol, Tween 20 and haloperidol were used. It was shown that the inclusion of haloperidol in the PAMAM dendrimer increased the solubility of haloperidol by 100 times. The results of preclinical studies show that the delivery system based on dendrimers with haloperidol made it possible to reduce the dosage of the drug administered intranasally by 6–7 compared to the intraperitoneal drug while maintaining therapeutic activity.

### 2.6. Advantages and Limitations of Intranasal Administration

As already noted, nasal dosage forms have a number of limitations. Firstly, they are characterized by relatively low dosages due to a relatively small absorption area; secondly, bioavailability may be limited due to the short residence time of the drug in the nasal cavity under the influence of mucociliary clearance. The most common liquid nasal forms require the use of preservatives to reduce the risk of their microbial contamination, which can negatively affect the mucous membranes, especially during a long course. In the case of dry nasal forms, there is a risk of particle aggregation during storage due to interaction with air moisture and surface phenomena. Nevertheless, due to the advantages of nasal dosage forms, more and more companies are showing interest in their development.

The use of the considered delivery systems should be justified primarily by achieving the target profile of the drugs being developed. It should be noted that nasal dosage forms for a number of APIs make it possible to avoid or significantly reduce the “first pass effect” that occurs for such drugs when administered orally or intravenously. As has been shown, the use of delivery systems in nasal dosage forms allows, in addition to this advantage, to solve the problems of increasing the bioavailability of the API (increasing solubility, adsorption, permeability), reducing the therapeutic effect onset time or increasing the action duration of the API’s targeted delivery.

Mucociliary clearance is a protective function of the body, which, in the case of using nasal dosage forms, does not allow the drug to have long-term contact with the nasal mucosa. The inclusion of micro- and nanoparticles in the composition and use of in situ gels containing mucoadhesive polymers, as well as thickeners, allows for the contact duration of the drug with the mucosa to be increased to several hours, which leads to higher adhesion rates. In addition, the ability to vary the physicochemical properties of the polymer matrix or in situ gel allows for controlling the rate of release of the API and achieving a longer therapeutic effect.

The use of solid lipid particles, micro- and nanoemulsions, and liposomes allows for some APIs to achieve higher permeability. It has been shown that emulsions, liposomes and dendrimers allow for a higher dosage due to better solubility of the API in them.

Much attention in research is paid to the delivery of the API directly to the central nervous system, bypassing the BBB. In all studies where the “nose to brain” pathway is discussed, the dosage form contains nano-objects. These can be polymer nanoparticles, solid lipid nanoparticles, liposomes, nanoemulsions, or dendrimer-based nanocarriers. Nevertheless, the considered micro-sized delivery systems (polymer microparticles, microemulsions, in situ gels without included nano-objects) can be successfully used in the treatment of local infections, such as the treatment of rhinitis.

The complication of the nasal formulation when using delivery systems leads to higher risks of toxicity, decreased drug stability, shorter shelf life and more stringent requirements for storage conditions, and complexity of dosing, especially for drugs that require long-term administration.

This review has shown a higher efficiency of the developed intranasal delivery systems in comparison with both traditional solutions and oral and injection forms. The advantages of nasal delivery over oral delivery are rapid absorption and high adsorption, which ensure a rapid onset of therapeutic action. This effect is associated with a large number of blood vessels in the nasal cavity and high blood supply. Additionally, with nasal delivery, there is no presystemic metabolism and degradation of the API in the gastrointestinal tract.

High bioavailability of small molecules is ensured, and the bioavailability of larger molecules is increased. Compared with oral and intravenous administration, nasal preparations require smaller doses of active substances. This reduces the risk of overdose and the occurrence of systemic side effects. Nasal administration leads to a decrease in the frequency of drug use and, importantly, minimizes the risk of resistance due to interruption of long-term treatment by the patient.

The nasal delivery method is non-invasive, convenient for patients, and can be used for nausea, vomiting, coma, fainting, and difficulty swallowing in children and the elderly.

Many successful developments of drugs are based on nanoscale delivery systems; examples of successful preclinical and clinical studies on the intranasal administration of such drugs are considered, which is confirmed by this review. Characteristics such as toxicity, safety and side effects can be limiting and critical during the launch of such drugs to the market.

## 3. Conclusions

A review of the scientific and technical literature showed that in the field of the development of nasal drugs, active research is underway to reformulate some therapeutic groups into a nasal form. The increased interest in this area is due to the fact that the nasal cavity has a unique set of anatomical characteristics for the delivery of active pharmaceutical ingredients. Intranasal delivery allows for non-invasiveness, rapid onset of action, and the absence of hepatic and intestinal metabolism. Today, the market offers nasal preparations of not only local, but also systemic action. An important task remains to improve the efficiency of this API delivery method. Various drug delivery systems are being researched and tested to optimize and improve drug adsorption. The article lists a wide range of drugs and describes the various dosage forms used in this delivery method.

## Figures and Tables

**Table 7:** Investigational nasal drugs for the treatment of Alzheimer’s disease.

API	Delivery System	Refs.
Rivastigmine	Nanoemulsion	[79]
	Microemulsion	[80,81]
	Gel in situ	[82]
Piperine	Nanoparticles	[83]
	Solid lipid particles	[84]
Insulin	Nanogel	[85]
Curcumin	Nanoparticles	[86]
	Gel in situ	[87]
	Microemulsion	[88]
Donzepil	Nanosuspension	[89]
	Liposomes	[90]
	Gel	[91,92]
	Microemulsion	[93]
Resveratrol	Gel in situ	[94]
	Solid lipid particles	[95]

**Table 8:** Investigational nasal drugs for the treatment of Parkinson’s disease.

API	Delivery System	Refs.
Celegiline	Chitosan nanoparticles	[98]
	Nanoemulsion	[99]
Bromocriptine	Chitosan nanoparticles	[100]
Rasagiline mesylate	Gel in situ	[101]
	Chitosan glutamate nanoparticles	[102]
	Chitosan-coated PLGA nanoparticles	[103]
Peonol	Gel in situ	[104]
Dopamine	Chitosan nanoparticles	[105]
	Borneol and lactoferrin co-modified nanoparticles	[106]
Pramipexole	Chitosan nanoparticles	[107]

**Table 9:** Investigational nasal antiepileptic drugs.

API	Delivery System	Refs.
Lamotrigine	Liposomes	[109]
	Nanoparticles	[110]
Letrozole	Nanoemulsion	[111]
Lorazepam	Gel in situ	[112]
Oxcarbazepine	Microemulsion	[113]
Phenytoin	Nanoemulsion	[114]

**Table 10:** Investigational nasal drugs for the treatment of schizophrenia.

API	Delivery System	Refs.
Quetiapine fumarate	Nanoemulsion	[118]
	Liposomes	[119]
Asenapine maleate	Nanoemulsion	[120]
	Gel in situ	[121]
Risperidone	Liposomes	[122]
Paliperidone	Microemulsion	[123,124,125]
	Gel in situ	[126]

**Table 11:** Investigational nasal medications for treating depression.

API	Delivery System	Refs.
Agomelatine	Gel in situ	[129]
	Microemulsion	[130]
Doxepin	Gel in situ	[131]
Duloxetine hydrochloride	Microemulsion	[132]
	Gel in situ	[133,134]
Fluoxetine hydrochloride	Gel in situ	[135]
Paroxetine	Nanoemulsion	[136]
	Gel in situ	[137]
Venlafaxine	Nanoparticles	[138,139]

**Table 12:** Investigational nasal vaccines.

API	Delivery System	Refs.
Avian influenza antigen (H5N1)	Gel in situ	[144]
Influenza A (H1N1) antigen	Particles	[145]
Norovirus virus-like particles	Particles	[146]
Shigellosis antigen	Spray	[147]
Hepatitis B antigen	Nanoparticles	[148,149]
Bovine serum albumin	Nanoparticles	[150]
Ovalbumin	Nanoparticles	[151]
*Clostridium botulinum* type	Nanoparticles	[152]
SARS-CoV-2 antigen	Microparticles	[153,154,155,156,157]
Tetanus toxoid	Nanoparticles	[158]
Recombinant antigens	Nanoparticles	[159]
*Brucella abortus* malate dehydrogenase antigen	Nanoparticles	[160]
Influenza A antigen (PR8)	Nanoparticles	[161]

**Table 13:** Polymers used to prepare particles for intranasal delivery systems.

Polymer Type	Polymer	Refs.
Natural polymers	Chitosan	[61,70,83,98,100,107,127,148,149,150,155,159,160]
	Chitosan derivatives	[49,102,105,157,158]
	Sodium alginate	[57,62,70]
Synthetic polymers	Copolymer of polylactic and glycolic acids (PLGA)	[106,110,154]
	Polylactide (or polylactic acid, PLA)	[66]
	Polycaprolactone	[86]

**Table 14:** Thermal- and ion-sensitive in situ gel systems for intranasal delivery.

Type of In Situ Gel	Reacting Agent	Refs.
Thermosensitive systems	Pluronic^®^ F-127	[32,33,129,133]
	Poloxamer 407	[36,82,91]
	Chitosan derivatives	[92,131,134,144]
Ion-sensitive systems	Xanthan gum	[61,175]
	Gellan gum	[63,87,94,112,135,137]
	Carbopol^®^, HPMC	[58,101,126]

## Data Availability

The data presented in this study are openly available in the article.
